# A *Tmprss2-CreER^T2^* Knock-In Mouse Model for Cancer Genetic Studies on Prostate and Colon

**DOI:** 10.1371/journal.pone.0161084

**Published:** 2016-08-18

**Authors:** Dong Gao, Yu Zhan, Wei Di, Amanda R. Moore, Jessica J. Sher, Youxin Guan, Shangqian Wang, Zeda Zhang, Devan A. Murphy, Charles L. Sawyers, Ping Chi, Yu Chen

**Affiliations:** 1 Key Laboratory of Systems Biology, Institute of Biochemistry and Cell Biology, Shanghai Institutes for Biological Sciences, Chinese Academy of Sciences, Shanghai, 200031, China; 2 Human Oncology and Pathogenesis Program, Memorial Sloan-Kettering Cancer Center, New York, New York, 10065, United States of America; 3 Weill Cornell Graduate School of Medical Sciences, Cornell University, New York, New York, United States of America; 4 Department of Medicine, Memorial Sloan-Kettering Cancer Center, New York, New York, 10065, United States of America; 5 Department of Medicine, Weill Cornell Medical College and New York–Presbyterian Hospital, New York, New York, 10065, United States of America; Thomas Jefferson University, UNITED STATES

## Abstract

Fusion between TMPRSS2 and ERG, placing ERG under the control of the TMPRSS2 promoter, is the most frequent genetic alteration in prostate cancer, present in 40–50% of cases. The fusion event is an early, if not initiating, event in prostate cancer, implicating the TMPRSS2-positive prostate epithelial cell as the cancer cell of origin in fusion-positive prostate cancer. To introduce genetic alterations into Tmprss2-positive cells in mice in a temporal-specific manner, we generated a *Tmprss2-CreER*^*T2*^ knock-in mouse. We found robust tamoxifen-dependent Cre activation in the prostate luminal cells but not basal epithelial cells, as well as epithelial cells of the bladder and gastrointestinal (GI) tract. The knock-in allele on the *Tmprss2* locus does not noticeably impact prostate, bladder, or gastrointestinal function. Deletion of Pten in Tmprss2-positive cells of adult mice generated neoplasia only in the prostate, while deletion of Apc in these cells generated neoplasia only in the GI tract. These results suggest that this new *Tmprss2-CreER*^*T2*^ mouse model will be a useful resource for genetic studies on prostate and colon.

## Introduction

The prostate epithelium is comprised of two distinct cell layers—an outer layer of basal cells in contact with the stroma and an inner layer of secretory luminal cells that produce constituents of the prostatic fluid. Prostate cancer shares many molecular and histologic similarities with luminal cells, including growth dependence on the androgen receptor (AR), as well as AR-dependent expression of seminal fluid proteases PSA and TMPRSS2. In contrast, prostate cancer seldom express basal markers and the absence of basal markers is a pathological criteria to diagnose prostate cancer.

Although most primary prostate cancers have luminal cell histology, the etiology and cell of origin of prostate cancer remain controversial. Early studies based on sphere formation *in vitro* and graft formation *in vivo* suggested that basal cells form the stem cells of the normal prostate and are the cells origin of prostate cancer [1,2]. However, recent studies using lineage tracing in genetically engineered mouse (GEM) models support the existence of a stem cell population in both the basal and luminal epithelia of the prostate [3,4] Within the luminal compartment, castration-resistant Nkx3-1-expressing cells (CARNs) have been shown to represent a source of stem cells that can regenerate the prostate epithelium and can be transformed when Pten is deleted [5]. Using the recently developed prostate 3D organoid culture system, we have shown that both single luminal and basal cells from either human or mouse prostates can give rise to organoids, implicating the existence of bipotential stem cells in each compartment [6,7]. Recent evidence also indicated that luminal cells are favored as cells of origin of prostate cancer [8].

Genomic fusion between the membrane-bound serine protease *TMPRSS2* and the ETS-family transcription factor *ERG* is an early genetic alteration occurring in ~50% of prostate cancers [9,10]. The fusion event results in ERG overexpression under the *TMPRSS2* promoter. These findings support the notion that *TMPRSS2*-expressing cells are important for prostate cancer initiation, and genomic alterations of these cells may trigger pathogenetic events. The identity of TMPRSS2-expressing cells is not fully elucidated. Different studies have reported that *Tmprss2* is preferentially expressed in basal cells, or luminal cells, or both [11,12,13].

In order to identify *Tmprss2* expressing cells, trace their lineage, and determine their tumorigenic capacity, w*e* generated a tamoxifen-inducible knock-in mouse model carrying the CreER^T2^ gene under the control of the *Tmprss2* promoter. We demonstrate the high efficiency of this model to selectively delete genes in the prostate luminal epithelium and colon epithelium. Furthermore, we show that conditional deletion of *Pten* and *Apc* in Tmprss2 expressing cells lead to prostate and colorectal transformation, respectively.

## Materials and Methods

### Generation of the *Tmprss2-CreER*^*T2*^ mouse

All mouse studies are approved by MSKCC Institutional Animal Care and Use Committee under protocol 11-12-027. Institutional guidelines for the proper, humane use of animals in research were followed.

To generate Tmprss2-FRT-NEO-FRT-CreER^T2^ targeting construct, we started with pRosa26PAm1 (a gift from Douglas Melton, Addgene #15036) [14,15], a targeting plasmid that contains PacI and AscI cloning sites between the 5’ and 3’ homology arms of the Rosa26 locus, followed by a diphtheria toxin cassette (DTA). We replaced the Rosa26 5’ arm with 3.15 kb fragment 5’ of exon 2 of the *Tmprss2* gene, generated by PCR using the 5’-TGG CTT CTG CTT CTG ATG-3’ and 5’-GCG TTA ATT AAG CCT TCA GCC TTC ACT TCA C-3’ primer pair on a mouse BAC clone, and cloned using PacI and SacII sites. We then replaced the Rosa26 3’ arm with a 5.05 kb fragment 3’ of exon 2 of the *Tmprss2* gene, generated by PCR using the 5’-GGG GCG CGC CTG GCC TTT TCC TTG TTC CT-3’ and 5’-GGG GGT CGA CAT GTG GCT CAG TGG TAA A-3’ primer pair, and cloned using AscI and SalI sites. PCR was performed using PFU turbo (Stratagene). We named the product pTmprss2PAm1.

Next, we took pBTG[14,15] (a gift from Douglas Melton, Addgene #15037), a plasmid with adenovirus splice acceptor (SA), followed by a LOX-STOP-LOX cassette, poly-linker site to insert the gene of interest, and IRES-nlsGFP, all between PacI and AscI sites, and performed the following two changes: 1) replaced LOX-STOP-LOX cassette with a FRT-Neo-FRT cassette (PCR amplified from pEZ-Frt-lox-DT (a gift from Klaus Rajewsky, Addgene #11736)) in the reverse direction and 2) cloned CreER^T2^ from pCAG-CreER^T2^ (a gift from Connie Cepko, Addgene #14797)[16] into the poly-linker site. We cloned the Frt-Neo-Frt-SA-CreER^T2^-IRES-nlsGFP into pTmprss2PAm1 to generate the targeting vector.

Gene targeting was performed at the Rockefeller University Gene Targeting Resource Center (Head: Chingwen Yang). The targeting plasmid was electrophoresed into albino C57BL/6J ES cells and G418 resistant clones were isolated by standard procedures. The clones were screened by Southern blotting using an external 3’ probe generated by PCR primers 5’-GTC ACC CCT CAC TGC ATT TT-3’ and 5’-ATG GAC ACT CCC AGG CTA GA-3’ cut by HindIII which gave a wild-type 7.5kb band and targeted 8.2kb band. Two positive clones were injected into C57BL/6J blastocysts by the MSKCC Mouse Genetics Core Facility (Head: Willie Mark), and chimeras were mated with albino C57BL/6J females. Germline transmission was confirmed in albino offspring using Southern blotting.

To excise the FRT-Neo-FRT cassette, we crossed the Tmprss2-FRT-NEO-FRT-CreER^T2^ mice with a FlpE-expressing mouse (B6;SJL-Tg(ACTFLPe)9205Dym/J, Jackson Laboratories) and excision was screened by Southern blotting using a 5’ probe generated by PCR primers 5’-GAT GGA GGC ATC TTT TCA CC-3’ and 5’-CCT CGC TGT CCC AAG ATT AC-3’ cut by EcoRI, which gave a wild-type band of 9.5kb, a targeted band of 9.9kb, and a targeted and FRT-Neo-FRT cassette excised band of 8.0kb. For subsequent generations, *Tmprss2-CreER*^*T2*^ mouse genotyping was performed by PCR of genomic DNA using the following primers: TMP2-11878F (5’-GGT GGG CTC TCC TGG CCA CA3’), TMP2-12186R(5’-TGC CAT CCT GCC T GT GTC AGC -3’), Cre-R4 (5’- CTC GTT GCA TCG ACC GGT AA-3’) with a wild-type band of 300 bp and targeted band of 380 bp.

### Mouse alleles

Apc^LoxP^ (APC^tm2Rak^) mice where exon 14 of *Apc* is flanked by LoxP sites [17] was a generous gift from Dr. Scott Lowe. Genotyping was performed using primers 5’-CAG ATG TCT TTA TGA GTT TGA-3’ and 5’-AGT GCT GTT TCT ATG AGT CAA C-3’ with a wild-type product of 388bp and LoxP allele of 498bp. To assess Apc deletion in tissue, we performed PCR on genomic DNA isolated from tissues using primers 5’-CAG ATG TCT TTA TGA GTT TGA-3’; 5’-AGT GCT GTT TCT ATG AGT CAA C-3’ and 5’-TTG GCA GAC TGT GTA TAT AAG C; the Apc LoxP and Apc deleted products were 498bp and 568 bp. Pten^LoxP^ mice (Pten^tm2.1Ppp^) in which exons 4–5 are flanked by LoxP sites [18], were used as previously described [10]. Ai3 (*B6*.*Cg-Gt(ROSA)26Sor*^*tm3(CAG-EYFP)Hze*^*/J*) mice of conditional CAG-driven YFP expression [19] and mT/mG (B6.129(Cg)-Gt(ROSA)26Sor^tm4(ACTB-tdTomato,-EGFP)Luo^/J) mice of membrane-targeted tdTomato and EGFP [20] and Actb-FlpE (Tg(ACTFLPe)9205Dym/J) that express the FlpE recombinase under the beta-actin promoter [21] were purchased from Jackson Laboratories.

### Mice tamoxifen treatment, sacrifice and tissue analysis

TY, TP and TA mice were injected with tamoxifen at 4mg/40g body weight using a 27-gauge needle and injected once every other day, a total of 3 times. Mice are euthanized by carbon dioxide asphyxiation as recommended by MSKCC Institutional Animal Care and Use Committee under protocol 11-12-027.

For histology and IHC, tissue was harvested from 10-week-old TY males, 22-week-old TP males and 12-week-old TA males. Tissues were fixed in 4% paraformaldehyde (w/v) at 4 degrees Celsius for 12 hours. Tissues were washed three times with cold PBS. All immunohistochemical and histological analyses were conducted by the MSKCC Molecular Cytology Core.

For immunofluorescence, tissues were fixed in 4% paraformaldehyde (w/v) at 4 degrees Celsius for 2 hours. Tissues were washed three times with cold PBS, cryopreserved by overnight incubation in 30% sucrose (w/w), frozen in OCT (Tissue Tek, Sakura Finetek) and sectioned.

### FACS and Quantive-RT-PCR

Single cell suspensions of TY mice anterior prostate were stained using CD326-PE/Cy7 (Biolegend; Clone# G8.8; 1:500) and DAPI. All the Q-PCR primers were purchased from Qiagen:

P63: PPM03458A-200
Ck5:PPM59967F-200
Ck14:PPM04519A-200
Ck8:PPM05184A-200
Ck18: PPM05184A-200

### Antibodies

The antibodies used for immunohistochemistry were pAKT Ser473 (Cell Signaling Technology; 4060; 1:50 dilution), PTEN (Cell Signaling Technology; 9188; 1:50 dilution), Beta-Catenin (BD Transduction Laboratories; 610154; 1:1000), Ki67 (Abcam; ab16667; 1:100) and GFP (Abcam; ab13970; 1:1000). P63 (Abcam; ab124762; 1:250) and Ck8 (Covance; MMS-162P; 1:1000) were used for immunofluorescence.

### Data mining of *TMPRSS2* expression

We obtained RNA-seq based *TMPRSS2* expression of human tissues from Genotype-Tissue Expression (GTEx) at www.gtexportal.org [22]. We obtained Arrymetrix MOE430 microarray based *Tmprss2* expression of mouse tissues from BioGPS [23].

## Results

In order to define *Tmprss2*-expressing cells in prostate, we generated an inducible *CreER*^*T2*^*-IRES-nlsGFP* mouse model under the control of the mouse *Tmprss2* gene promoter. At baseline levels, nuclear GFP expression marks *Tmprss2*-expressing cells and the Tmprss2-positive lineage can be traced and genetically manipulated when crossed with LoxP-lines and exposed to tamoxifen.

**Fig 1:**
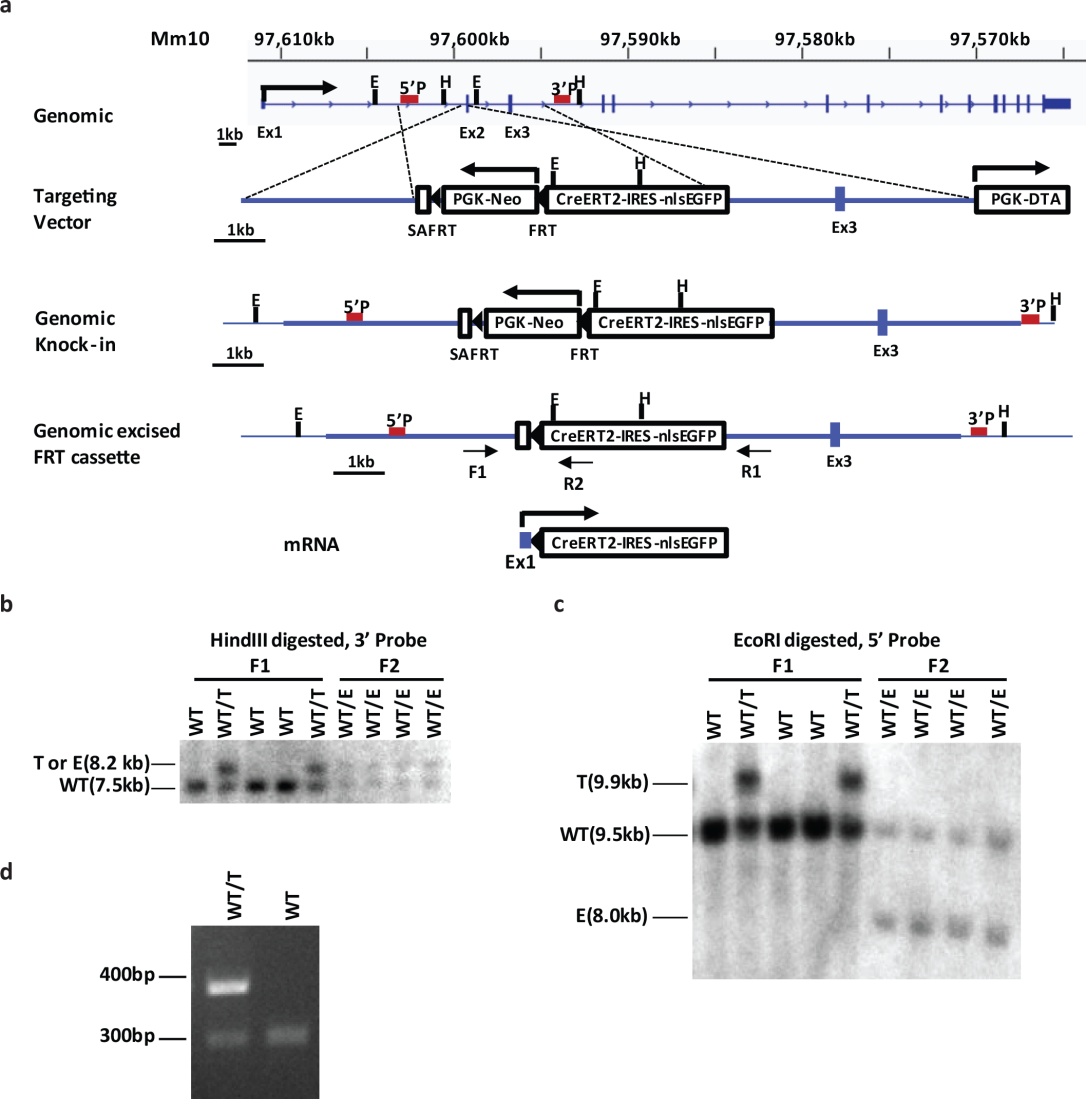
Generation of the *Tmprss2-CreER*^*T2*^ knock-in mouse. (a) Schematic of targeting strategy. A cassette including an adenovirus splice acceptor (SA), followed by PGK-driven neomycin selection cassette flanked by FRT recombination sites, followed by the CreER^T2^-IRES-nlsEGFP was used to replace exon 2 of mouse Tmprss2. The cassette is flanked by 3.5kb 5’ and 5kb 3’ homology arms. 5’ and 3’ Southern probes as was as HindIII (H) and EcoRI (E) sites and genotyping PCR primers (universal F1, wild-type specific R1, and knock-in specific R2) are depicted. The final transcript includes the non-coding exon 1 of Tmprss2 spliced into the CreER^T2^-IRES-nlsEGFP gene. (b) Southern blot using 3’ probe and HindIII digestion. WT mice give a 7.5 kb band and the targeted mice (regardless of neomycin cassette) give a 8.2 kb band. (c) Southern blot using 5’ probe and EcoRI digestion. WT mice give a 9.5 kb band; the targeted mice with neomycin cassette (T) give a 9.9 kb band, while the mice with excised neomycin cassette (E) give a 9.5 kb band. (d) Genotype determination of wild-type and heterozygous mice by PCR. Wild-type fragment is 300-bp and mutant is 380-bp.

In human prostate cancer, the first exon of *TMPRSS2* is entirely within the non-coding 5’ UTR and most *TMPRSS2-ERG* fusions involve the first intron of *TMPRSS2*, with the resulting fusion transcript containing no coding sequence of *TMPRSS2*. We therefore replaced exon 2 with a cassette including an adenovirus splice acceptor (SA), followed by a PGK-driven neomycin selection cassette flanked by FRT recombination sites, followed by the CreER^T2^-IRES-nlsGFP. After excision of the neomycin cassette, the mouse should express a chimeric transcript containing exon1 of *Tmprss2* as the 5’ UTR followed by CreER^T2^ cDNA (**Fig 1A**).

After homologous recombination in embryonic stem cells and germline transmission, we observed two independent lines with the correct integration verified by Southern blot analysis using both 5’ and 3’ probes (**Fig 1B**). We next crossed F1 mice with Actb-FlpE mice and Southern blot analysis using the 5’ probe confirmed excision of the neomycin cassette in all FlpE and CreER^T2^ double-positive F2 mice (**Fig 1C**). Genotyping for the *Tmprss2-CreER*^*T2*^ allele was performed by polymerase chain reaction (PCR), resulting in a 380-bp product for the knock-in allele and a 300-bp product for wild-type (**Fig 1A and 1D**). Homozygous *Tmprss2-CreER*^*T2*^ had no visible phenotype and were generated at Mendelian ratio, consistent with prior observation that *Tmprss2* knockout mice had no phenotype [24].

**Fig 2:**
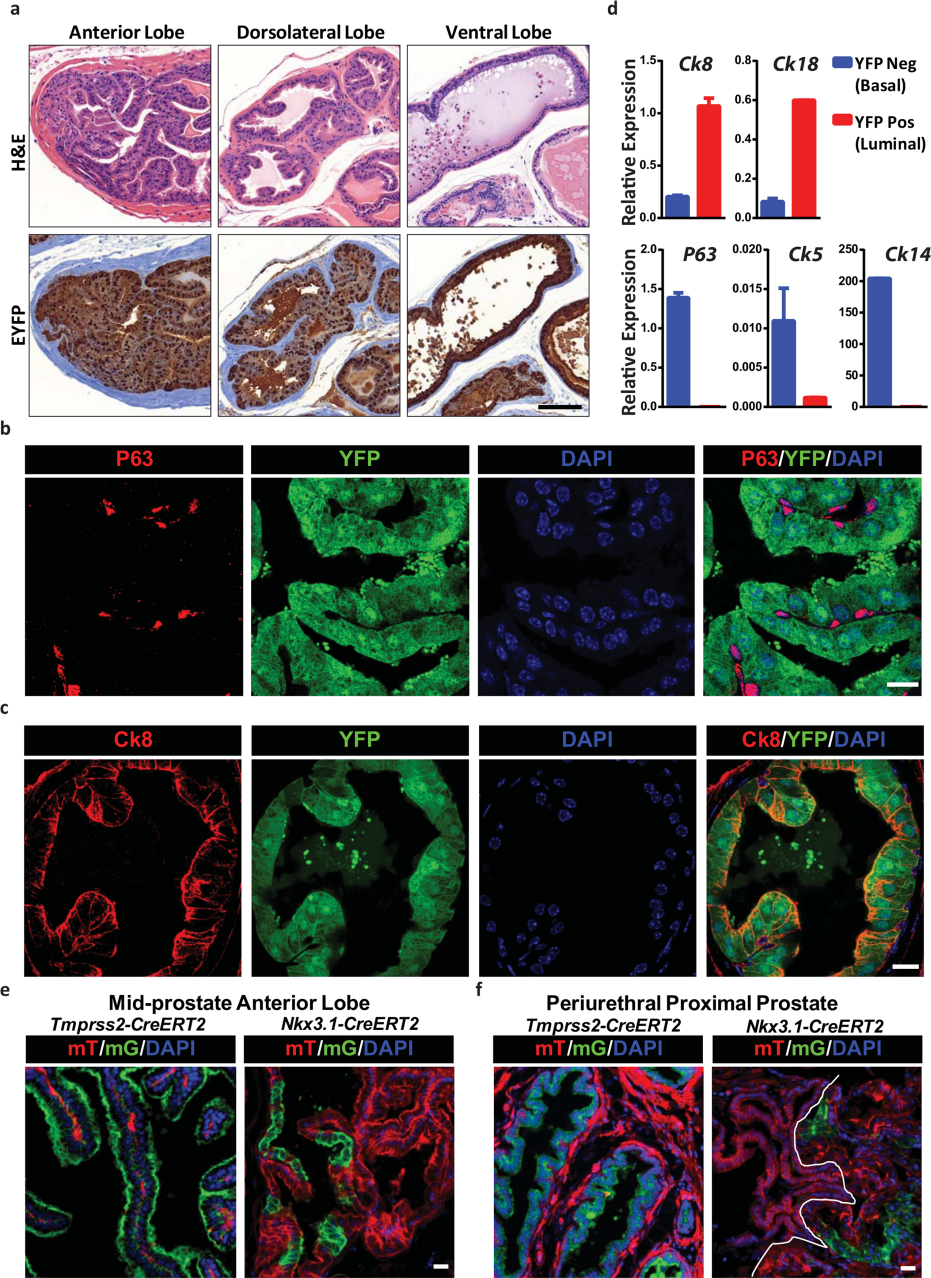
Prostate specific activity of *Tmprss2-CreER*^*T2*^ mice. (a) H&E and YFP IHC of anterior prostate, dorsolateral prostate, ventral prostate in TY mice. Scale bar represents 100 μM. (b) IF stain of basal cell marker P63 with endogenous YFP and DAPI fluorescence in TY mice. Scale bar represents 20 μM. (c) IF stain of luminal cell marker Ck8 with endogenous YFP and DAPI fluorescence in TY mice. Scale bar represents 20 μM. (d) Quantitative RT-PCR analysis of basal (*Ck5*, *Ck14*, *P63*) and luminal (*Ck8*, *Ck18*) marker expression in YFP+ and YFP- epithelial cells. Basal cell markers are strongly expressed YFP- cells; luminal cell markers strongly expressed in YFP+ cells. Expression was normalized to Actin. Results are shown as mean ± SD. (e) Comparison *of Tmprss2-CreER*^*T2*^ with *Nkx3*.*1-CreER*^*T2*^ driven conversion of membrane tdTomato (mT) to membrane EGFP (mG) of the anterior prostate. Scale bar represents 50 μM. (f) Same as in (e) but in periurethral proximal prostate. These cells are tightly packed with scant cytoplasm. The white line in the *Nkx3*.*1-CreER*^*T2*^ mouse separates the anterior prostate from periurethral prostate. Scale bar represents 50 μM.

The nuclear GFP signal of *CreER*^*T2*^*-IRES-nlsGFP* is dim at baseline levels and is difficult for direct lineage trace. To test the efficiency and specificity of the novel knock-in line, we crossed *Tmprss2-CreER*^*T2*^ mice with Rosa26-EYFP mice with a CAG driven YFP Cre-reporter [19]. We examined the YFP expression pattern in the prostate gland of 10-week-old male *Tmprss2-CreER*^*T2*^; *Rosa26-EYFP* /*EYFP* (TY) mice after 2 weeks of tamoxifen treatment. YFP immunohistochemistry (IHC) showed that labeling was highly efficient, and the majority of the prostate epithelium appeared labeled (**Fig 2A**). Analysis of direct YFP fluorescence combined with immunofluorescence (IF) against luminal marker Cytokeratin 8 (Ck8) or basal maker p63 showed that only the Ck8 positive luminal cells exhibited YFP signal, while the p63 positive basal cells and stroma cells were negative for YFP in the anterior, dorsolateral and ventral lobes of the prostate (**Fig 2B and 2C**). Quantification of YFP-expressing cells indicated that in the anterior and dorsolateral lobes, approaching 100% of luminal epithelial cells (n = 635) were positive and in the ventral lobe, approximately 80% of luminal cells (n = 476) were positive. We separated YFP-positive and YFP-negative prostate epithelia cells from TY mice anterior prostate tissue using florescence activated cell sorting (FACS). Consistent with our histological observation, the YFP-positive population had high luminal-cell-specific gene expression, *Ck8* and *Ck18*, while the YFP-negative population had high basal-cell-specific gene expression, *P63*, *Ck5* and *Ck14* (**Fig 2D**).We compared the *Tmprss2-CreER*^*T2*^ recombinase activity with that of *Nkx3-1-CreER*^*T2*^, a commonly used inducible Cre-driver for prostate luminal cells. We crossed both lines to *Rosa26-mT/mG*, a mouse that expresses membrane-targeted tdTomato (mT) at baseline- and membrane-targeted GFP (mG) after excision [20] and found two important differences that highlight the utility of the *Tmprss2-CreER*^*T2*^ mouse model. First, the efficiency of recombination within prostate luminal cells is much higher in *Tmprss2-CreER*^*T2*^ (99.6%, n = 234) compared to *Nkx3-1-CreER*^*T2*^ (23.7%, n = 223). Second, within the proximal peri-urethral prostate, which is comprised of more compact epithelial cells and is thought to have a greater number of stem cells, *Tmprss2-CreER*^*T2*^ is highly active (97.7%, n = 215), whereas *Nkx3-1-CreER*^*T2*^ lacks activity (1.04%, n = 192) (**Fig 2E and 2F**).

**Fig 3:**
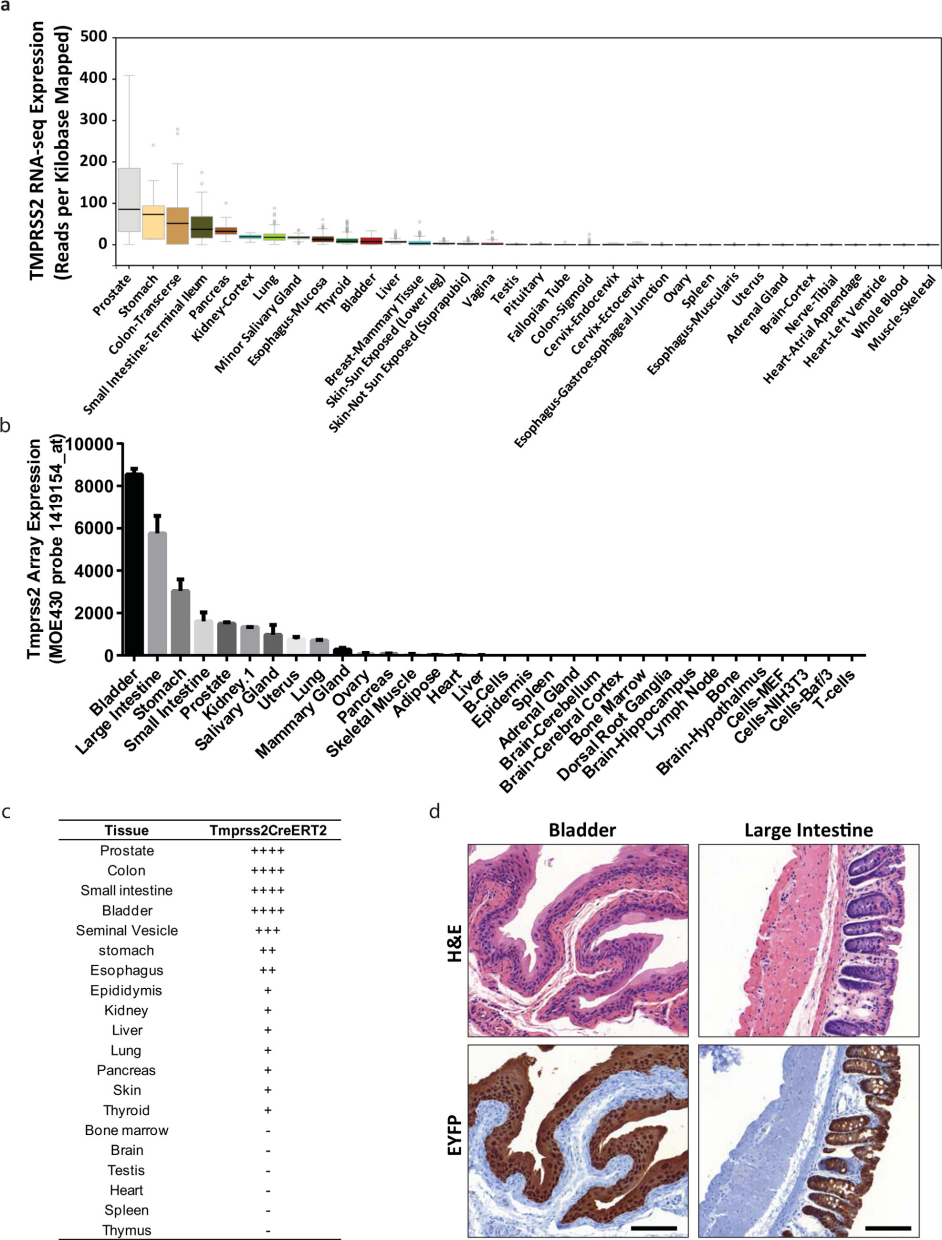
Tissue distribution of *Tmprss2-CreER*^*T2*^ mediated recombination. (a) RNA-seq-based expression of *Tmprss2* mRNA in human tissues from the Genotype-Tissue Expression (GTEx) project. (b) MOE430-based expression of *Tmprss2* mRNA in mouse tissues from BioGPS. (c) Table of recombination efficiency, based on YFP IHC, in tissues of TY mice after tamoxifen administration. (d) H&E and YFP IHC of bladder and colon in TY mice. Scale bars represent 100 μM.

Mining of normal tissue gene expression data showed that *TMPRSS2* is not prostate-specific and is expressed in multiple organs, specifically of the GI tract in both human and mouse (**Fig 3A and 3B**). Notably, TMPRSS2 expression also have difference between human and mouse, such as bladder, which has relatively high expression in mouse, but it appears to be quite low in human. To determine the tissue specificity of CreER^T2^ activity beyond the prostate, nineteen other tissues, namely the testis, epididymis, seminal vesicle, pancreas, colon, small intestine, stomach, esophagus, bladder, bone marrow, brain, heart, kidney, liver, lung, skin, spleen, thymus and thyroid were collected from male TY mice and analyzed for YFP staining. We found that almost all the epithelial cells of colon and bladder were positive for YFP staining, while there were no detectable YFP positive stroma cells in these organs (**Fig 3C and 3D**). Recombination was also observed in epididymis, seminal vesicle, pancreas, small intestine, stomach, esophagus, kidney, liver, lung, skin and thyroid (**Fig 3C**).

**Fig 4:**
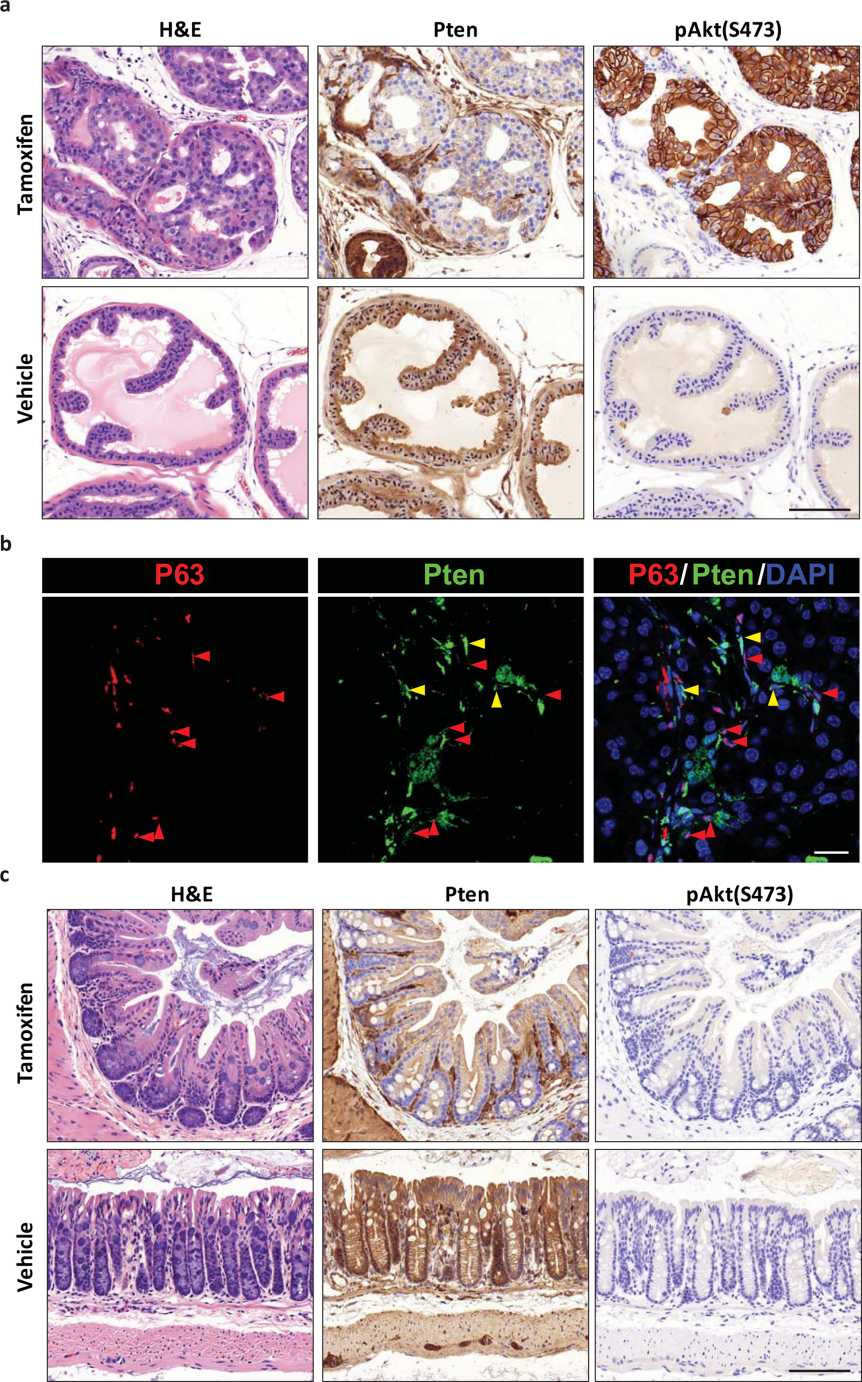
Tissue-specific tumorigenesis of Pten deletion. (a) H&E and Pten, pAkt(S473) IHC of prostate from TP mouse following tamoxifen or vehicle administration. Scale bar represents 100 μM. (b) IF stain of basal cell marker P63 with Pten and DAPI fluorescence in TP mice. Red arrows and yellow arrows indicate P63+ basal cell and P63- stromal cells. Scale bar represents 25 μM. (c) H&E and Pten, pAkt(S473) IHC of prostate from TP mouse following tamoxifen or vehicle administration. Scale bar represents 100 μM.

We asked whether *Tmprss2-CreER*^*T2*^ is active in the tumor initiating cells and if *Tmprss2-CreER*^*T2*^-mediated deletion of tumor suppressor genes can generate tissue specific tumorigenesis. First, we analyzed deletion of *Pten*, a tumor suppressor implicated in prostate cancer initiation. To achieve *Pten* deletion, we crossed *Pten*^*LoxP/LoxP*^ mice [18] to the *Tmprss2-CreER*^*T2*^ knock-in line. We treated the *Tmprss2-CreER*^*T2/ T2*^*; Pten*^*LoxP/LoxP*^ (TP) mice with tamoxifen at 8 weeks of age and analyzed the prostate and colon 12 weeks later. Hematoxylin and eosin (H&E) staining of the prostate showed prevalent prostatic intraepithelial neoplasia (mPin), with cribriform growth and enlarged nuclei in tamoxifen but not vehicle injected mice (**Fig 4A**). IHC and IF staining of PTEN and P63 showed that Pten loss is specific to the prostate luminal epithelial cells, while Pten is expressed in some prostate basal epithelial cells and stromal cells surrounding the prostate acini (**Fig 4A and 4B**). The loss of Pten corresponds with an increase of AKT phosphorylation in tamoxifen treated prostate luminal cells. We next analyzed colonic epithelium in these TP mice and found no visible gross or histological abnormalities. IHC analysis showed robust loss of Pten throughout the colonic epithelium. However, AKT phosphorylation was not detectable using the same staining method as the prostate pAKT staining, suggesting very low baseline PI-3 kinase activity that cannot induce AKT phosphorylation despite loss of Pten (**Fig 4C**).

**Fig 5:**
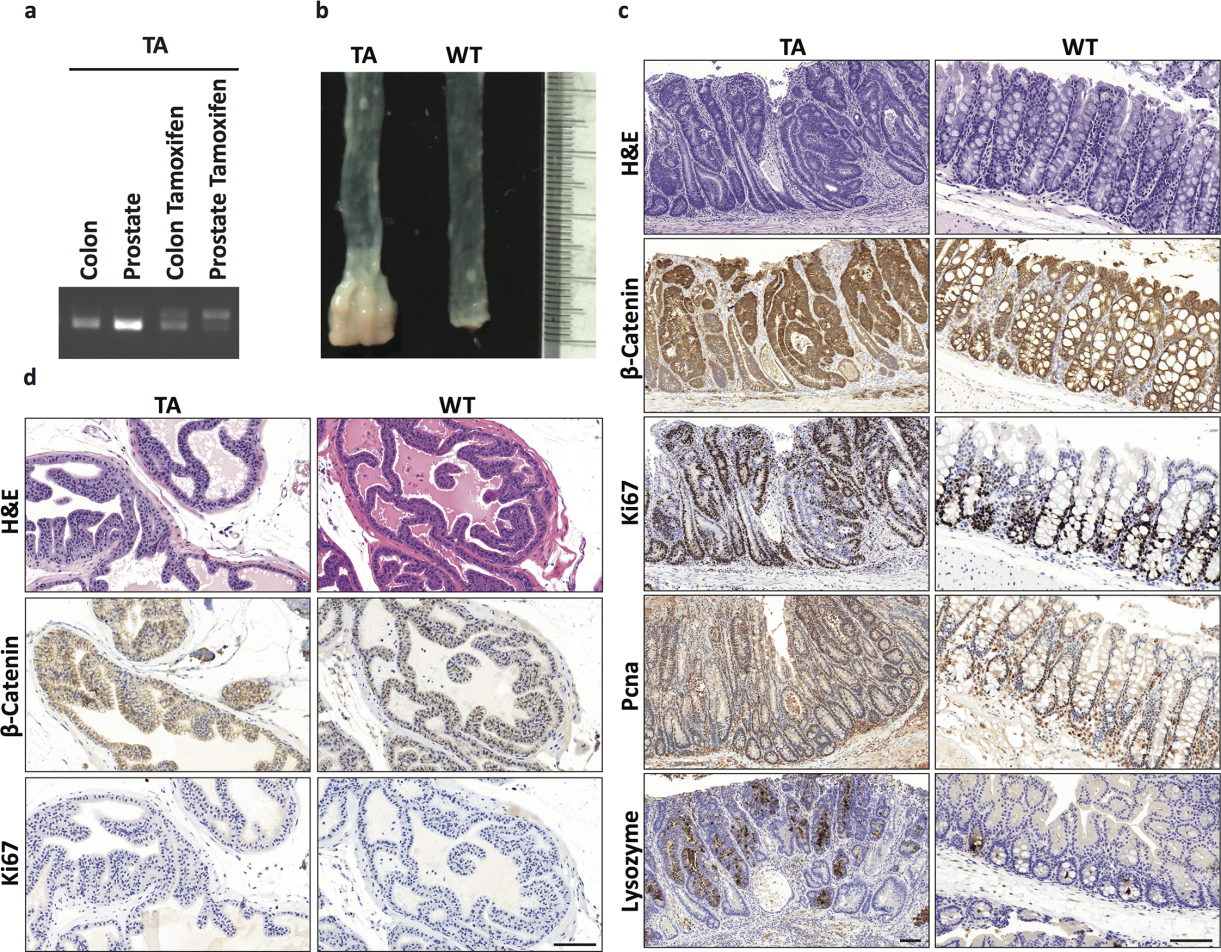
Tissue specific tumorigenesis of Apc deletion. (a) Characterization of Cre-mediated recombination in Tam-treated *Tmprss2CreER*^*T2/ T2*^*; Apc*^*LoxP/LoxP*^ (TA) mice. Genomic DNA isolated from the indicated organs of a Tam-treated mouse were analyzed by PCR. The positions of the *Apc* floxed 498bp, and recombined 568bp DNA segments are indicated. (b) Gross pathology of colon of TA and wild-type mice following tamoxifen administration showing macroscopic colon polyps in tamoxifen treated mice. (c) H&E and β-catenin, Ki67 stain of TA and wild-type mouse colon. (d) H&E and β-catenin, Ki67 stain of TA and wild-type mouse prostate. Scale bars represent 100 μM.

We next analyzed deletion of Apc, a gatekeeper in colorectal cancer tumorigenesis. We crossed *Apc*^*LoxP/LoxP*^ mice to the *Tmprss2-CreER*^*T2*^ mice and treated the *Tmprss2-CreER*^*T2/T2*^*; Apc*^*LoxP/LoxP*^ (TA) mice with tamoxifen at 8 weeks of age and analyzed the mice colon and prostate 12 weeks post tamoxifen treatment. PCR of genomic DNA isolated from prostate and colon showed the presence of PCR product specific for Apc-deletion only in tamoxifen-treated mice (**Fig 5A**). The colonic epithelium of tamoxifen treated TA mice showed features characteristic of Apc-loss adenomas (**Fig 5B**). The loss of Apc in these mice corresponds to an increase in β-catenin staining and marked hyperproliferation with increased Ki67 and Pcna staining outside the crypts, characteristic of differentiation arrest by the identification of lysozyme-positive Paneth cells outside the base of crypt (**Fig 5C**). Although the prostate had loss of Apc (**Fig 5A**), the prostate exhibited normal histology (**Fig 5D**). IHC showed a subtle increase in membrane-localized β-catenin levels in cells and no change in Ki67 index (**Fig 5D**).

## Discussion

GEM models of cancer have provided important insights into cancer initiation and progression, and have important implications for clinical studies and clinical trials. Various mouse lines were generated over recent years by selectively introducing targeted mutations in prostatic epithelium through Cre recombinase under the control of the mouse mammary tumor virus promoter MMTV-Cre [25], a modified probasin promoter PB-Cre4 [26], or the prostate-specific antigen (PSA) promoter PSA-Cre [27,28]. More recently, several prostate-specific CreER^T2^ mouse models, including knock-in to the *Nkx3-1* locus [5], transgenic under the human PSA promoter [29] and transgenic under a modified probasin promoter (ARR2Pb) [30] have been generated, each able to induce mPin when crossed to *Pten*^*LoxP/LoxP*^ mice. This *Tmprss2-CreER*^*T2*^ knock-in strain is highly efficient and allows precise control over the timing of introducing gene alterations in the mouse prostate and colon, and accurately mimics the late onset of human prostate cancer and colon cancer.

Here, we show that *Tmprss2-CreER*^*T2*^ is expressed in several organs and rapidly induces prostate neoplasia after induction of Pten deletion and colorectal neoplasia after induction of Apc deletion. We further find that the effects of oncogenic activation are surprisingly tissue-specific and recapitulate that of human cancer. In human prostate cancer, *PTEN* loss is an early tumorigenic lesion, while Wnt-pathway activation through aberrations in *APC*, *CTNNB1*, *RNF43*, and *RSPO2* are found in metastatic castration-resistant cancer, suggesting they are associated with tumor progression [31,32]. In human colorectal cancer, Wnt-pathway activation is a gatekeeper event while mutations of loss of *PTEN* rarely occur.

In conclusion, we have established a new CreER^T2^ knock-in mouse line, and these knock-in mice can be used to investigate Tmprss2-expressing cells and their descendant cells at various stages *in vivo*. We believe that the generated knock-in mice in this article could be useful for studying the initiation and progression of prostate and colon cancers.
